# Organic Trace Minerals Enhance the Gut Health of British Shorthair Cats by Regulating the Structure of Intestinal Microbiota

**DOI:** 10.3390/metabo14090494

**Published:** 2024-09-11

**Authors:** Yingyue Cui, Mingrui Zhang, Haotian Wang, Tong Yu, Anxuan Zhang, Gang Lin, Yuhan Guo, Yi Wu

**Affiliations:** 1 State Key Laboratory of Animal Nutrition and Feeding, College of Animal Science and Technology, China Agricultural University, Beijing 100193, China; sy20233040846@cau.edu.cn (Y.C.); zhangmingrui@cau.edu.cn (M.Z.); wanghaotian@cau.edu.cn (H.W.); 2022304010128@cau.edu.cn (T.Y.); shubai929@cau.edu.cn (A.Z.); 2 Beijing Alltech Biological Products (China) Co., Ltd., Beijing 100600, China; glin@alltech.com (G.L.); bguo@alltech.com (Y.G.)

**Keywords:** trace mineral sources, gut microbiota, gut microbial metabolites, immunity, adult cats

## Abstract

**Simple Summary:**

At present, studies have shown that organic trace minerals have a positive impact on animal health compared with inorganic trace minerals. However, the effect of organic trace minerals on the intestinal health of adult cats has not been extensively explored. In our study, we investigated the effects of different trace mineral sources on the gut microbiota and digestive health in British Shorthair cats and dietary organic trace minerals on the intestinal barrier, immune function, anti-inflammatory ability, gut microbiota, and metabolites in adult British Shorthair cats. Our results suggest that compared to inorganic trace minerals, dietary organic trace minerals could enhance immune function, improve inflammatory state, and regulate the gut microbiota structure in cats.

**Abstract:**

Trace minerals are essential for biological processes, including enzyme function, immune response, and hormone synthesis. The study assessed the effects of different dietary trace minerals on the gut health, microbiota composition, and immune function of cats. Eighteen adult British Shorthair cats were divided into three groups receiving inorganic trace minerals (ITM), a 50/50 mix of inorganic and organic trace minerals (ITM + OTM), or organic trace minerals (OTM) for 28 days. The OTM showed enhanced immune capacities, reduced intestinal barrier function, and lower inflammation condition. The OTM altered gut microbiota diversity, with a lower Simpson index and higher Shannon index (*p* < 0.05). Specifically, the abundance of Bacteroidota, Lachnospiraceae, and Prevotella in the OTM group were higher than the ITM group (*p* < 0.05). Metabolomic analysis identified 504 differential metabolites between the OTM and ITM groups (*p* < 0.05, VIP-pred-OPLS-DA > 1), affecting pathways related to steroid hormone biosynthesis and glycerophospholipid metabolism (*p* < 0.05, VIP-pred-OPLS-DA > 2). Additionally, there was a significant correlation between intestinal microbiota and differential metabolites. To conclude, dietary OTM can modulate the gut metabolite and microbiota composition, enhance immune and intestinal barrier function, and mitigate inflammation in cats, highlighting the benefit of using OTM in feline diet to promote the intestinal and overall health.

## 1. Introduction

Trace minerals are integral to numerous biological processes, including enzyme function, immune response, and the synthesis of hormones [1]. Zinc, for example, is recognized as a crucial mineral for immune function due to its unique interaction and collaboration with multiple minerals of the immune system, and it is essential for DNA synthesis and cell division [2]. Copper is an essential mineral that constitutes numerous structural proteins with enzymatic functions, such as superoxide dismutase, metallothionein, and ceruloplasmin. Furthermore, it evinces modulation of immune processes and has a hematopoietic effect [3]. Selenium acts as an antioxidant, thereby safeguarding cells from oxidative damage [4]. The absorption and bioavailability of these trace minerals can significantly influence their efficacy in maintaining animal health [5].

Trace minerals in inorganic form are typically available in forms such as sulfates, oxides, and chlorides [6]. These inorganic trace minerals (ITMs) are commonly used in commercial cat diet because of their cost efficiency and stability. However, the antagonistic interactions in the gastrointestinal tract can impede the absorption of ITMs, thereby decreasing their bioavailability [7]. For example, zinc oxide and zinc sulfate, which are the common inorganic zinc sources, have been shown to have limited absorption in the animal gastrointestinal tract [8]. Conversely, organic trace minerals (OTMs) are bonded to organic molecules, peptides, or amino acids, for example, forming complexes like zinc methionine or copper proteinate [6]. These organic forms are designed to mimic the natural minerals found in food and to enhance their absorption and bioavailability [9]. One study demonstrated that trace minerals derived from organic sources can maintain poultry performance and improve egg shell quality [10]. Another research showed that compared with inorganic selenium, organic selenium not only had higher bioavailability but also increased daily weight gain, improved thyroid metabolism, and reduced the level of pro-inflammatory factors in puppies [11]. In addition, organic selenium also showed a probiotic effect, which helped to stabilize the intestinal microbiota of puppies [11].

Cats play an important role in human life. Therefore, more and more pet owners are beginning to pay attention to the health of cats. Gut health is a critical aspect of overall health in cats, influencing nutrient absorption and immune function [12]. The gastrointestinal tract is home to a complicated community of microorganisms, unitedly identified as gut microbiota, which can not only provide energy for the intestinal tract through metabolites and regulate the digestive and metabolic functions but also improve the immune state and resist intestinal pathogens [13]. Imbalanced intestinal microbiota, known as dysbiosis, can result in inflammatory bowel disease, diarrhea, and other gastrointestinal disorders [14].

Both inorganic and organic trace minerals can be used as nutritional supplements in diets, but their impacts on intestinal health may differ significantly. ITMs can sometimes disrupt the gut microbiota or cause irritation to the gastrointestinal tract when administered in high concentrations. For example, the use of OTMs to completely replace ITMs in the prenatal and postpartum foods of pregnant cows enhanced rumen fermentation, energy metabolism, and production performance with higher dry matter intake and lower non-esterified fatty acid circulation levels [15]. Another study showed that compared with ITMs, OTMs can improve the antioxidant capacity of fattening pigs, which was manifested by the increase in serum superoxide dismutase activity and the decrease in malondialdehyde level [16]. OTMs, on the other hand, may offer benefits beyond their basic nutritional value. Due to their higher bioavailability, they are more efficiently absorbed and utilized by the body, potentially reducing the risk of negative interactions within the gut [9]. Moreover, it was demonstrated that OTMs are capable of modulating the immune response, maintaining the integrity of the gut lining, and promoting a healthy gut microbiota balance [17].

Despite extensive prior investigation in farm animals, research comparing the impact of inorganic and organic trace mineral sources on gut health and gut microbiota in pets is inadequate. As a consequence, the objective of this study was to examine the effects of OTMs in replacing ITMs on gut health in adult cats, with a particular emphasis on the gut microbiota composition and its metabolites.

## 2. Materials and Methods

### 2.1. Animals and Experimental Treatments

The experimental protocol for dietary treatment and animal treatment was approved by the Institutional Animal Care and Use Committee of China Agricultural University, and the experiment identification code was AW50503202-2-5.

Prior to the commencement of the study, all British Shorthair cats underwent medical examinations to assess their suitability for participation in the trial. These checks included fecal scoring, body condition scoring, and body weight (BW) and parasite analysis. All indicators were normal, and no cats had parasitic infections. The animals had received no relevant drugs or diets, nor undergone antibiotics, immunosuppressive treatments, or surgical procedures in the past three months prior to the commencement of the study. No cats exhibited evidence of allergies, immune-mediated disease, or chronic gastrointestinal issues. Cats unable to ingest oral food, suffering from chronic medical conditions, or pregnant or nursing were excluded from the study. All cats were maintained in the same feeding environment with a consistent basal diet meeting the adult cat nutritional requirements required by NRC (2006), as detailed in Table 1. Ultimately, eighteen healthy cats participated fully in the trial.

**Table 1:** Ingredient and nutrient composition of the basal diets.

Items	Content
Ingredient, %	
Chicken powder	49.80
Fish powder	3.21
Rice bran	20.00
Fish oil	2.00
Chicken oil	3.00
Potato starch	1.00
Tapioca flour	0.60
Taurine	19.00
Salt	0.20
Vitamin ^1^	0.49
Mineral premix ^2^	0.40
Total	0.30
Nutrient composition	
Metabolic energy, kcal/kg	4031
Phosphorus, %	1.52
Calcium, %	1.72
Ether extract, %	17.66
Crude protein, %	41.01

^1^ Per kilogram of feed, the vitamin premix provided vitamin A (15,000 IU), vitamin B1 (30 mg), vitamin B2 (28 mg), vitamin B3 (110 mg), vitamin B5 (85 mg), vitamin B6 (12 mg), vitamin B12 (0.19 mg), vitamin D3 (15 IU), and vitamin E (75,300 IU). ^2^ The composition of each group of mineral premix is shown in Table 2.

**Table 2:** The composition and content of trace minerals.

Treatments	The Composition and Content of Trace Minerals, mg/kg DM				
	Zn	Fe	Cu	Mn	Se
ITM	ZnSO_4_, 75	Fe_2_(SO_4_)_3_, 80	CuSO_4_, 5	MnSO_4_, 7.6	Na_2_SeO_3_, 0.3
ITM + OTM	ZnSO_4_, 37.5 Bioplex ^1^ Zn, 37.5	Fe_2_(SO_4_)_3_, 40 Bioplex Fe, 40	CuSO_4_, 2.5 Bioplex Cu, 2.5	MnSO_4_, 3.8 Bioplex Mn, 3.8	Na_2_SeO_3_, 0.15 Sel-Plex ^2^ 2000, 0.15
OTM	Bioplex Zn, 75	Bioplex Fe, 80	Bioplex Cu, 5	Bioplex Mn, 7.6	Sel-Plex 2000, 0.3

^1^ Bioplex: Bioplex minerals are trace minerals bonded to amino acids and various peptides; ^2^ Sel-Plex: Sel-Plex^®^ is Alltech’s proprietary organic form of selenium yeast.

### 2.2. Experimental Design and Sample Collection

Eighteen adult British Shorthair cats (nine females and nine males; mean BW 3.74 ± 0.11 kg, mean age 1.50 ± 0.50 years) participated in the experiment. During a 7-day observation period, cats had a consistent basal diet and were raised in the same living environment. After a 7-day observation period, a 28-day experimental period was conducted, during which the dietary treatments were as follows: (1) ITM group (control; group 1): In the diet, all sources of trace minerals are inorganic. (2) ITM + OTM group (group 2): In the diet, the mineral premix contained 50% inorganic and 50% organic trace minerals. (3) OTM group (group 3): In the diet, all sources of trace minerals are organic. All trace minerals were supplied by Alltech, Inc. The composition and content of trace minerals in each treatment group are shown in Table 2.

The BW of each cat was recorded on days 0 and 28. On d 0 and d 28, blood samples of 2 mL were drawn from the small saphenous vein located on the lateral side of each cat’s hind limb. The samples were rested in centrifuge tubes at indoor temperature for 35~60 min for coagulation stratification and then centrifuged for 10 min at 4 °C at 3000 rpm to separate serum. The serum samples were retained at −20 °C for the subsequent determination of immune, inflammatory, and intestinal barrier function indicators. On day 28, all fecal samples were collected multiple times a day, and all fecal samples of the same cat were preserved in sterile tubes and reserved at −80 °C for microbiota and metabolomics analyses. On day 28, the fecal score and feed consumption of each cat were recorded. The fecal scoring criteria are shown in Table 3.

### 2.3. Serum Parameters Measurement

Serum immune indexes included immunoglobulin (Ig)A, IgM, and IgG. Intestinal barrier function indicators included diamine oxidase (DAO), intestinal fatty acid-binding protein (i-FABP), zonulin, and lipopolysaccharide (LPS). Inflammation indicators included interleukin-1β (IL-1β), interleukin-6 (IL-6), tumor necrosis factor-α (TNF-α), histamine, calprotectin, α-1 antitrypsin (A1AT), and monocyte chemotactic protein-1 (MCP-1). All these indexes were analyzed using ELISA kits from Shanghai Enzyme-linked Biotechnology Co., Ltd. (Shanghai, China), with the analysis conducted according to the instructions supplied by the manufacturer. Data reading was performed using a spectrophotometer from BioTek Instruments, Inc. ((Synergy™ H4), Beijing Branch, Beijing, China).

### 2.4. Microbiota Analysis

Total bacterial DNA from fecal samples were extracted by the QIAamp Fast DNA Stool Mini Kit (Qiagen, Hilden, Germany), using universal primers 338F (5’-ACTCCTACGGGAGGCAGCAG-3′) and 806R (5′-GGACTACHVGGGTWTCTAAT-3’) to amplify the V3–V4 region of 16S rRNA, which were subsequently pooled in equimolar amounts. The resulting amplicons were sequenced on the Illumina MiSeq platform, generating paired-end reads of 300 base pairs (bp). The raw 16S rRNA sequences’ quality control was performed with fastp software (version 0.19.6), and splicing was carried out with access to FLASH software (version 1.2.11). Operational taxonomic units (OTUs) were created from optimized sequences at a 97% sequence similarity level with UPARSE software (Version 11). Systematics for each OTU characteristic sequence were analyzed using the RDP Classifier (Version 2.2), which was cross-referenced with the Silva 138 database. The confidence threshold was set at 0.7. Community composition at each taxonomic level was confirmed, and in abundance between the groups, the Kruskal–Wallis H test was employed to identify significant discrepancies. Alpha diversity indices, namely the Simpson index and the Shannon index, were computed using the Mothur software (Version 1.30.2), while beta diversity was assessed through Bray–Curtis distance and visualized via principal component analysis (PCA).

### 2.5. Metabolomics Analysis

Next, 50 mg fecal samples were placed in a 2 mL centrifuge tube, accompanied by a 6 mm grinding bead. For metabolite extraction, 0.4 mL of leach solvent containing methanol and water (*v*:*v* = 4:1), with 20 μg/mL L-2-chlorophenylalanine as internal standard, was added. The samples were ground for 6 min in a frozen tissue grinder (Wonbio-96c, Shanghai Wanbo Biotechnology Co., Ltd., Shanghai, China) at −10 °C and a frequency of 50 Hz, and then, low-temperature ultrasonic extraction at a temperature of 5 °C and a frequency of 40 kHz was carried out for 30 min. After being placed at −20 °C for 30 min, the samples were subjected to centrifuge at 13,000× *g* and 4 °C for 15 min. For subsequent LC/MS analysis, the supernatant was then migrated to injection vials.

The non-targeted metabolomic analysis of fecal matter was conducted at Majorbio Bio-Pharm Technology Co., Ltd. (Shanghai, China) by means of the ACQUITY HSS T3 column (Waters, Milford, MA, USA) and the Thermo UHPLC-Q Exactive HF-X system. Progenesis QI software (Waters Corporation, Milford, MA, USA) was employed to initially process the LC/MS raw data. The data matrix was subjected to a series of processes aimed at eliminating internal standard peaks and other false-positive peaks, comprising those resulting from column bleed, noise, and derivatized reagent peaks. This involved a process of de-redundancy and peak pooling. Metabolites were identified through a search of relevant databases, including the Majorbio Database, HMDB (http://www.hmdb.ca/ (accessed on 13 July 2024)), and Metlin (https://metlin.scripps.edu/ (accessed on 12 July 2024)), and were uploaded the data matrix to the Majorbio cloud platform (https://cloud.majorbio.com (accessed on 10 July 2024)) for further analysis. Orthogonal partial least squares–discriminant analysis (OPLS-DA) and principal component analysis (PCA) were performed via the R package “ropls” (Version 1.6.2), and assessed model stability through a 7-cycle interactive validation. Metabolites with a *p*-value fewer than 0.05 and a variable importance in projection (VIP) value larger than 1 were identified as differential metabolites on the basis of the *p*-value calculated through the Student’s *t*-test and the VIP value generated through the OPLS-DA model.

### 2.6. Statistical Analysis

IBM SPSS Statistics 26 (Chicago, IL, USA) was used for data analysis, and GraphPad Prism (version 8.3.0, San Diego, CA, USA) was used to visualize the data.

The R tool was used to plot the microbial sequencing data. The R vegan package was used to generate the heatmaps, and bacterial community bar graphs were created by the R ggplot package. The Kruskal–Wallis H test was employed to ascertain the relative abundance of the microbiota. The remaining data were subjected to one-way ANOVA with the Tukey post hoc test. Statistical significance was defined as *p* < 0.05. The data are expressed as the mean ± standard error of the mean (SEM).

## 3. Results

### 3.1. Growth Performance

The effects of various treatments on the growth performance and average fecal score of cats on day 28 are shown in Table 4. No statistically significant differences were discovered in the BW (body weight) and ADG (average daily gain) of cats among the three treatments on days 0 and 28 (*p* ≥ 0.05). Similarly, among the three groups, no statistically significant differences were observed in the fecal score (*p* ≥ 0.05).

### 3.2. Immune Indexes

**Figure 1:**
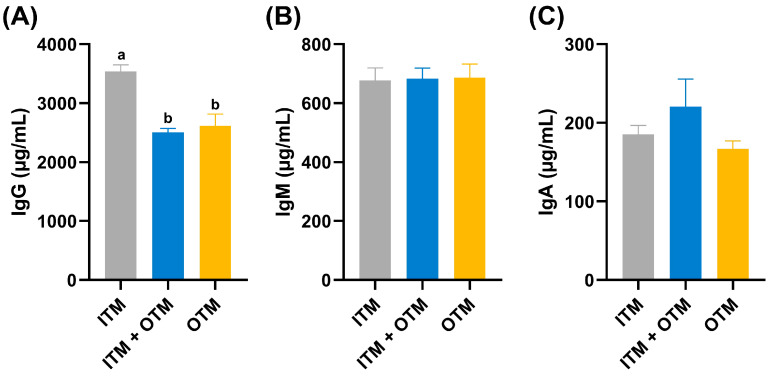
Influences on dietary trace minerals from different sources on serum immune indexes of cats. (**A**) IgG; (**B**) IgM; (**C**) IgA. (1) ITM group: In the basal diet, the mineral premix was inorganic. (2) ITM + OTM group: In the basal diet, the mineral premix included 50% inorganic trace minerals and 50% organic trace minerals. (3) OTM group: In the basal diet, the mineral premix was organic; *n* = 6. ^a,b^. Different superscript letters show significant difference among the three groups (*p* < 0.05), and there is no significant difference among the three groups without different superscript letters (*p* ≥ 0.05).

Figure 1 presents the immunoglobulin parameters of cats exposed to various treatments. Compared with ITM group, the IgG levels significantly decreased in cats from the OTM and ITM + OTM groups (*p* < 0.05), and the IgG level was not significantly different between the ITM + OTM and OTM groups (*p* ≥ 0.05; Figure 1A). However, among the three groups, the IgM and IgA levels showed no significant differences (*p* ≥ 0.05; Figure 1B,C).

### 3.3. Inflammatory Factors

**Figure 2:**
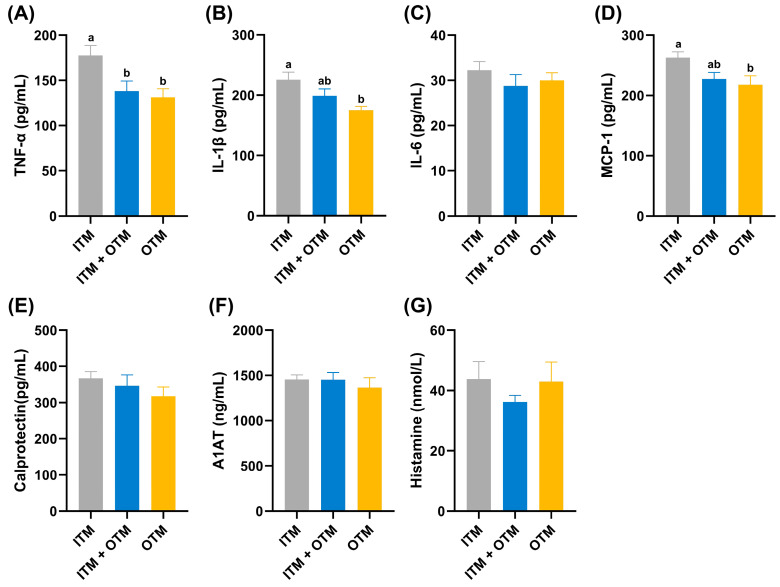
Effects of dietary trace minerals from different sources on serum inflammatory factors of cats in the different groups. (**A**) TNF-α; (**B**) IL-1β; (**C**) IL-6; (**D**) MCP-1; (**E**) calprotectin; (**F**) A1AT; (**G**) histamine. (1) ITM group: In the basal diet, the mineral premix was inorganic. (2) ITM + OTM group: In the basal diet, the mineral premix included 50% inorganic trace minerals and 50% organic trace minerals. (3) OTM group: In the basal diet, the mineral premix was organic; *n* = 6. ^a,b^. Different superscript letters show significant difference among the three groups (*p* < 0.05), and there is no significant difference among the three groups without different superscript letters (*p* ≥ 0.05).

Figure 2 showed the inflammatory factors of cats in the different treatments. Among the inflammatory factors, TNF-α concentration was significantly higher in the cats on the ITM diet compared to the cats on the TIM + OTM or OTM diets (*p* < 0.05; Figure 2A). Additionally, the cats on the ITM diet showed significantly higher concentrations of IL-1β and MCP-1 compared to the cats on the OTM diet, while the concentrations of these indices showed no significant difference in the cats fed the ITM + OTM diet (*p* < 0.05; Figure 2B,D). The levels of IL-6, calprotectin, A1AT, and histamine did not differ significantly among the three dietary treatment groups (*p* ≥ 0.05; Figure 2E–G).

### 3.4. Intestinal Permeability Parameters

**Figure 3:**
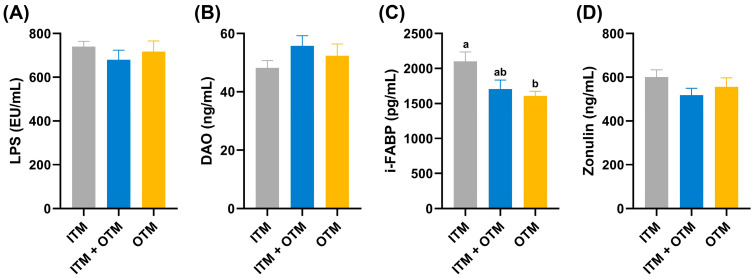
Effects of dietary trace minerals from different sources on intestinal barrier function parameters of cats in the different groups. (**A**) LPS; (**B**) DAO; (**C**) i-FABP; (**D**) zonulin. (1) ITM group: In the basal diet, the mineral premix was inorganic. (2) ITM + OTM group: In the basal diet, the mineral premix included 50% inorganic trace minerals and 50% organic trace minerals. (3) OTM group: In the basal diet, the mineral premix was organic; *n* = 6. ^a,b^. Different superscript letters show significant difference among the three groups (*p* < 0.05), and there is no significant difference among the three groups without different superscript letters (*p* ≥ 0.05).

Figure 3 presents the intestinal permeability parameters across the various dietary treatment groups. The cats on the ITM diet showed a significantly decreased concentration of i-FABP compared to the cats on the OTM diet (*p* < 0.05; Figure 3C). There was no significant difference in the concentrations of DAO, LPS, and zonulin among the three diet groups (*p* ≥ 0.05; Figure 3A,B,D).

### 3.5. The Richness and Diversity of Fecal Microbiota

**Figure 4:**
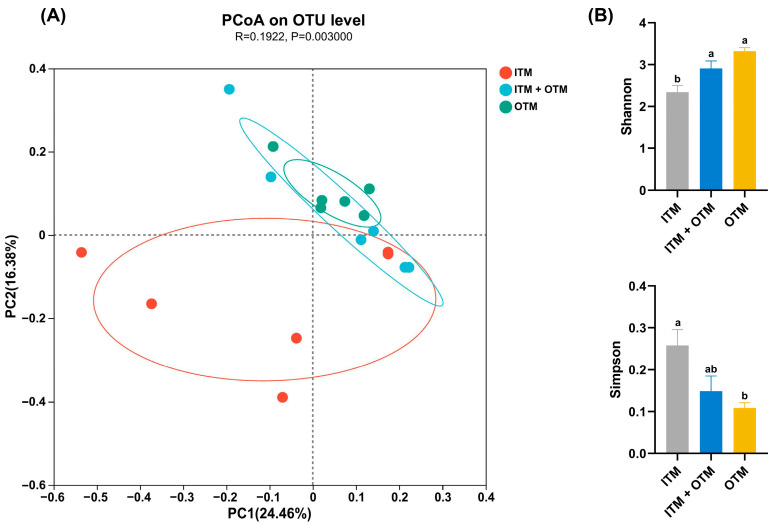
The impact of different treatments on the microbial diversity in feces of cats. (**A**) Principal component analysis at the OTU level. (**B**) The α-diversity analysis manifested as the Shannon and Simpson indices. (1) ITM group: In the basal diet, the mineral premix was inorganic. (2) ITM + OTM group: In the basal diet, the mineral premix included 50% inorganic trace minerals and 50% organic trace minerals. (3) OTM group: In the basal diet, the mineral premix was organic; *n* = 6. ^a,b^. Different superscript letters show significant difference among the three groups (*p* < 0.05), and there is no significant difference among the three groups without different superscript letters (*p* ≥ 0.05).

PCoA demonstrated distinct fecal microbial communities clustering at the OTU level for each group (*p* < 0.05; Figure 4A). In the cats that consumed the ITM diet, a lower Shannon index was exhibited compared to the cats that consumed the OTM and ITM + OTM diets (*p* < 0.05; Figure 4B). Additionally, compared to the cats that consumed the OTM diet, the Simpson index of the cats that consumed the ITM diet was significantly increased (*p* < 0.05; Figure 4B), indicating that trace minerals from organic sources could raise the diversity and richness of microbiota.

### 3.6. Fecal Microbiota Composition

**Figure 5:**
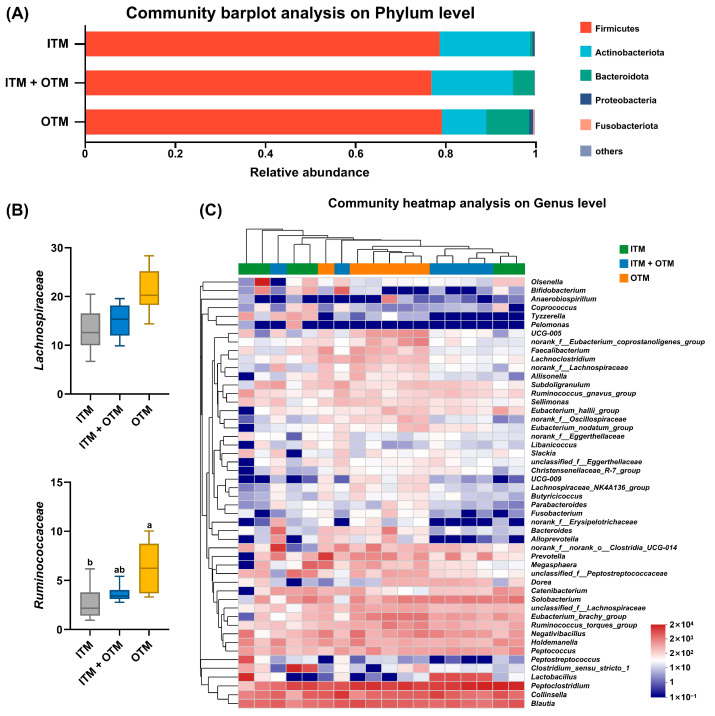
Shifts in the fecal microbiota composition in cats fed various treatments. (**A**) Relative abundance of fecal microbiota at the phylum level. (**B**) Relative abundance of *Lachnospirceae* and *Ruminococcaceae*. (**C**) Relative abundance of fecal microbiota at the genus level. (1) ITM group: In the basal diet, the mineral premix was inorganic. (2) ITM + OTM group: In the basal diet, the mineral premix included 50% inorganic trace minerals and 50% organic trace minerals. (3) OTM group: In the basal diet, the mineral premix was organic; *n* = 6. ^a,b^. Different superscript letters show significant difference among the three groups (*p* < 0.05), and there is no significant difference among the three groups without different superscript letters (*p* ≥ 0.05).

The presence of fecal bacteria was identified at different levels, including the phylum, family, and genus, with notable differences in abundance observed among the various treatments. The OTM group had a higher abundance of Bacteroidota (Figure 5A) in their fecal microbiota compared to the ITM group at the phylum level (*p* < 0.05). At the family level, the abundance of Lachnospiraceae and Ruminococcaceae was lower in the cats that consumed the ITM diet than that in the cats that consumed the OTM diet (*p* < 0.05; Figure 5B). At the genus level, the abundance of *Eubacterium_brachy_group* and *Prevotella* in the OTM group showed an increase (*p* < 0.05; Figure 5C) compared to the ITM group. Compared to the cats fed the OTM diet, the abundance of *UCG-005*, *norank_f__Eubacterium_coprostanoligenes_group*, *Faecalibacterium*, and *Lachnoclostridium* was decreased in the cats fed the ITM and ITM + OTM diets (*p* < 0.05). However, the abundance of *Lactobacillus* in the ITM + OTM group was higher (*p* < 0.05) than that in the OTM group, and it showed no significant difference in the ITM group compared to the OTM and ITM + OTM groups (*p* ≥ 0.05).

### 3.7. Fecal Metabolomics Composition

**Figure 6:**
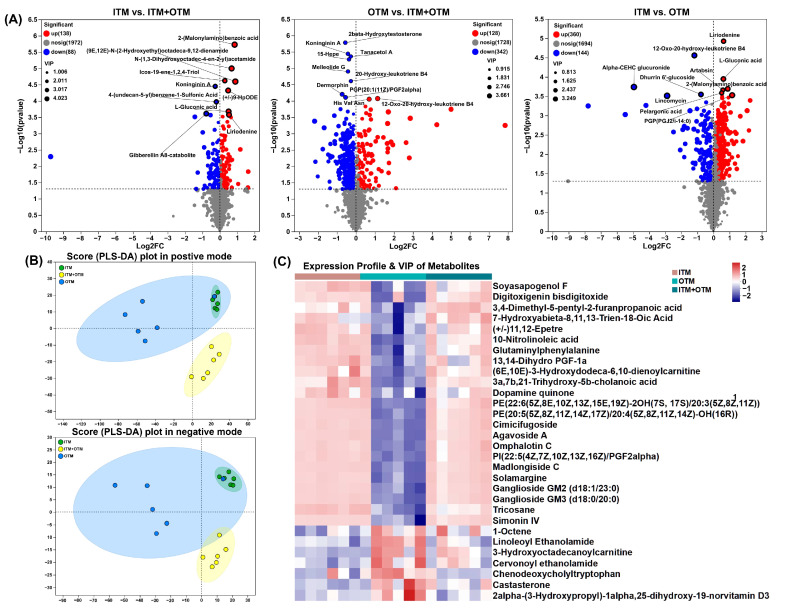
Changes in the fecal metabolites of cats subjected to different treatments. (**A**) Volcano plot of differential metabolites in ITM vs. ITM + OTM, OTM vs. ITM + OTM, and ITM vs. OTM (VIP-pred-OPLS-DA > 1, *p* < 0.05). (**B**) Scores scatter plot for differential metabolites in ITM, OTM, and ITM + OTM group as recognized through PLS-DA in both positive and negative modes. (**C**) Analysis of differential metabolites was enforced in the three groups using VIP value assessment and a clustering heat map. (1) ITM group: In the basal diet, the mineral premix was inorganic. (2) ITM + OTM group: In the basal diet, the mineral premix included 50% inorganic trace minerals and 50% organic trace minerals. (3) OTM group: In the basal diet, the mineral premix was organic; *n* = 6.

In order to ascertain the impact of altered metabolic pathways on the treatment of diverse trace mineral sources, a non-targeted metabolomics assay was conducted on feces. Figure 6 shows the differential metabolites between different groups. A total of 226 different metabolites (VIP-pred-OPLS-DA > 1, *p* < 0.05) were identified in ITM vs. ITM + OTM, of which 138 were up-regulated, and 88 were down-regulated. In OTM vs. ITM + OTM, 342 down-regulated differential metabolites and 128 up-regulated differential metabolites were identified, totaling 470 (*p* < 0.05, VIP-pred-OPLS-DA > 1). A total of 504 differential metabolites were identified in ITM vs. OTM, with 144 down-regulated and 360 up-regulated (VIP-pred-OPLS-DA > 1, *p* < 0.05). The datasets were analyzed using PLS-DA plots in both positive and negative modes, which showed that the metabolites profiles of the three groups were distinctly separated (Figure 6B). Compared to the OTM group, The VIP score, calculated using the OPLS-DA model, indicated that 87 metabolites exhibited VIP > 2 (Figure 6C), including up-regulated agavoside A (VIP = 2.63), madlongiside C (VIP = 2.62), ganglioside GM3 (d18:0/20:0) (VIP = 2.60), simonin IV (VIP = 2.58), ganglioside GM2 (d18:1/23:0) (VIP = 2.44), and solamargine (VIP = 2.40) in the ITM group. Meanwhile, compared to the ITM group, the concentrations of 3-hydroxyoctadecanoylcarnitine (VIP = 2.45), cervonoyl ethanolamide (VIP = 2.31), linoleoyl ethanolamide (VIP = 2.28), and chenodeoxycholyltryptophan (VIP = 2.12) were up-regulated in the OTM group. In the ITM + OTM group, the VIP scores of agavoside A (VIP = 2.84), madlongiside C (VIP = 2.80), ganglioside GM3 (d18:0/20:0) (VIP = 2.73), simonin IV (VIP = 2.72), ganglioside GM2 (d18:1/23:0) (VIP = 2.64), solamargine (VIP = 2.59), and PE(20:5(5Z,8Z,11Z,14Z,17Z)/20:4(5Z,8Z,11Z,14Z)-OH(16R)) (VIP = 2.32) were greater than those in the OTM group and were up-regulated in the ITM + OTM group.

**Figure 7:**
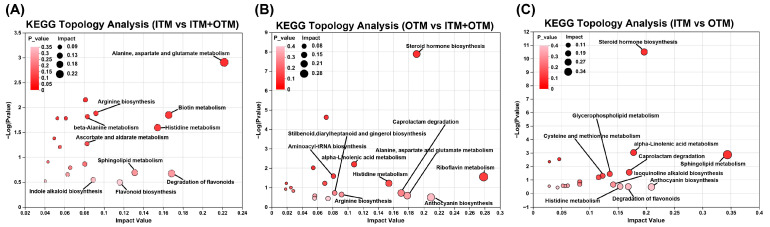
KEGG topology analysis for differential metabolites. (**A**) ITM vs. ITM + OTM; (**B**) OTM vs. ITM + OTM; (**C**) ITM vs. OTM; (1) ITM group: In the basal diet, the mineral premix was inorganic. (2) ITM + OTM group: In the basal diet, the mineral premix included 50% inorganic trace minerals and 50% organic trace minerals. (3) OTM group: In the basal diet, the mineral premix was organic; *n* = 6.

Using the high-quality KEGG metabolic pathways as a reference knowledge base, the pathway topology analysis was performed (Figure 7). Ten significantly disturbed pathways with higher pathway impact scores (>0.1) and lower *p*-values were found between the ITM and OTM groups based on the confirmed metabolites and their concentration changes. The pathways included steroid hormone biosynthesis, caprolactam degradation, isoquinoline alkaloid biosynthesis, cysteine and methionine metabolism, alpha-linolenic acid metabolism, and glycerophospholipid metabolism, and they were not identified in ITM vs. ITM + OTM. Seven perturbed metabolic pathways exhibited a higher pathway impact (>0.1) and lower *p*-values between the OTM and ITM + OTM groups, such as riboflavin metabolism, steroid hormone biosynthesis, alpha-linolenic acid metabolism, histidine metabolism, caprolactam degradation, anthocyanin biosynthesis, aspartate and glutamate metabolism, and alanine. These data demonstrated the significant changes in metabolites following different trace mineral source treatments.

### 3.8. Correlation Analysis between Differential Metabolites and Genus Level Gut Microbiota

**Figure 8:**
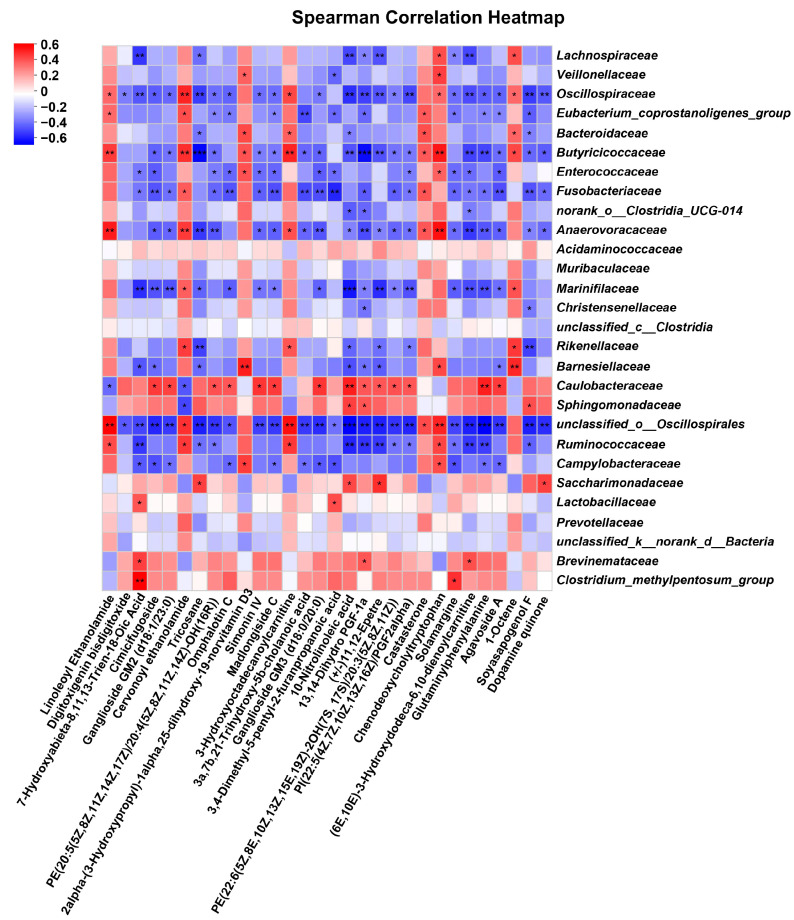
Using the Spearman correlation analysis to analyze the correlation between the differentiated genus and differential metabolites. * *p* < 0.05; ** *p* < 0.01; *** *p* < 0.001. (1) ITM group: In the basal diet, the mineral premix was inorganic. (2) ITM + OTM group: In the basal diet, the mineral premix included 50% inorganic trace minerals and 50% organic trace minerals. (3) OTM group: In the basal diet, the mineral premix was organic; *n* = 6.

To determine whether the observed alternations in differential metabolites were associated with microbial activity, we conducted a Spearman correlation analysis for assessing the correlation between the differential metabolites and the differentiated genera. Subsequently, we identified the top 30 metabolites that exhibited the strongest correlations (Figure 8). The findings revealed a significant association between certain gut microbiota and differential metabolites at the genus level, such as *Clostridium_methylpentosum_group*, *unclassified_o__Oscillospirales*, *Anaerovoracaceae*, *Butyricicoccaceae*, *Barnesiellaceae*, and *Erysipelotrichaceae*.

## 4. Discussion

Trace minerals are essential for growth and metabolism. They participate in numerous physiological functions within the animal body, such as enzyme activity, reduction in oxidative stress, and nutrient metabolism [18]. However, studies have shown that excessive intake of inorganic trace minerals can be harmful to animals [7,19]. In contrast, OTMs are more beneficial to animal gut and overall health [9,20]. OTMs are usually formed by binding to amino acids, proteins, or organic acids to form organic chelates or complexes. This structure makes trace minerals more stable in the digestive tract and more bioavailable, which can reduce the risk of toxicity and strengthen the immune system [6]. However, there is little research about the influences of the sources of trace minerals on cats. Therefore, this study examined the impacts of different trace mineral sources on the gut health in adult British Shorthair cats.

Immunoglobulins are a class of globular proteins with antibody activity or chemical structure, and they are essential to the animal immune system. Immunoglobulins can activate the complement system, elicit inflammatory responses and cytotoxicity, and promote the activation and proliferation of macrophages and natural killer cells, thereby enhancing the immune response [21]. Trace minerals can reduce the mRNA expression level of pro-inflammatory factor and increase the contents of anti-inflammatory factor and immunoglobulin in the inflammatory response [22]. IgG is the predominant component of immunoglobulins, comprising approximately 75% of the total immunoglobulin content in serum [23]. Replacing ITMs with OTMs at lower levels significantly increased the serum IgG levels in weaned piglets [24]. Supplementation of OTMs to sows during pregnancy and lactation can increase the levels of IgM, IgG, and IgA in piglet serum and sow milk [25]. However, one study showed that supplementation of laying hens with dietary organic micronutrients significantly reduced serum IgG levels [10]. Similarly, in this study, IgG levels showed a significant decrease in cats fed with the ITM + OTM and OTM diets compared to the ITM group. In fact, research has demonstrated that cats with chronic gingivostomatitis had significantly increased serum levels of IgG, IgM, and IgA, and the salivary levels of IgG, IgM, and albumin were higher [26]. Despite the absence of a statistically significant difference in IgM and IgA levels across the various groups in this study, the trend of IgG suggests a potential for OTMs to influence broader aspects of immune function, and more research is warranted to gain more scientific information regarding the effect of mineral sources on immunoglobulins in cats.

The intestine, which is regarded as the largest immune organ, can influence the immune system to a great extent. Cytokines, which are endogenous polypeptides primarily produced by immune system cells, mediate diverse immune responses, exerting a multitude of biological effects. The balance between anti-inflammatory and pro-inflammatory cytokines is pivotal for maintaining the body’s robust physiological functions and immune status. IL-1β and TNF-α can induce leukocyte production and activation, enhance local and systemic inflammatory responses, and have strong pro-inflammatory effects [27]. A study showed that supplementation of rabbits with selenomethionine for 21 days restrained the T-2 toxin-induced rise in the levels of IL-1β, TNF-α, and IL-6 in serum, thus alleviating the inflammatory response [28]. In addition, the serum levels of IL-1β, TNF-α, and IL-6 showed a significant decrease for rats fed dietary OTMs, which alleviated arthritis symptoms in rats [29]. IL-1β and TNF-α can bind to tumor necrosis factor receptor or IL-1 receptor on the surface of target cells and then activate the mitogen-activated protein kinase pathway and nuclear factor κB (NF-κB) pathway, ultimately inducing the concentration of MCP-1 [30,31]. Chemokines such as MCP-1 can regulate the progression of a wide range of pathological changes through the infiltration and migration of inflammatory cells along with other cytokines to the site of inflammation [32]. A study showed that organic selenium significantly reduced the level of MCP-1 induced by mammary tumors in mice [33]. In our study, compared to the cats fed the ITM diet, the levels of pro-inflammatory cytokines including MCP-1, TNF-α, and IL-1β in the cats fed the OTM diet exhibited a significant decrease. These reductions indicated an alleviated inflammatory response due to the organic form of minerals.

The intestinal epithelial cells constitute a physical barrier through tight junctions and the mucus layer, which prevents harmful substances such as pathogens and toxins from entering the body [34]. A study showed that low-molecular-weight seleno-aminopolysaccharide contributed to a reduction in intestinal damage and permeability in weanling rats, as evidenced by a decrease in DAO, D-LA, and LPS levels [35]. Another study showed that feeding ducks with organic zinc glycine chelate for 35 days significantly improved intestinal morphology and promoted the expression of tight junction proteins compared to inorganic zinc [36]. In our study, we assessed intestinal barrier function by measuring levels of LPS, DAO, i-FABP, and zonulin in cat serum. LPS, which is involved in the formation of the cell wall of Gram-negative bacteria, elicited an increase in the levels of pro-inflammatory factors and gut barrier permeability [37]. DAO is a biological enzyme responsible for metabolizing and degrading dietary biogenic amines, and damage or reduction in epithelial cells can lead to a decrease in DAO level [38,39]. Zonulin is the only known physiological regulator of intestinal permeability. Binding of zonulin to receptors such as protease-activated receptor 2 on the surface of intestinal epithelial cells can activate intracellular signaling cascade pathways. The activation of these pathways triggers remodeling of the cytoskeleton, leading to alterations in the location and function of tight junction proteins [40,41,42]. I-FABP is mainly found in the cytoplasm of small-intestinal epithelial cells. When the cells are damaged or necrotic, i-FABP is released into the bloodstream at a rapid rate [43,44], so the serum level of i-FABP can be used to assess the extent of gut barrier damage. The lack of significant differences in zonulin, DAO, and LPS levels among the groups might be due to the short duration of the study. Nevertheless, in our study, the i-FABP level was lower in cats that consumed the OTM diet compared with cats that consumed the ITM diet, indicating that dietary OTMs improved intestinal barrier integrity in cats and supported better intestinal health.

Gut microbiota and their metabolites can greatly influence the health and disease status of animals [13,45]. Cats are specialized carnivores, and their dietary structure is different from that of other mammals, which determines their highly diverse and complex intestinal ecosystems. The gut microbiota of cats has been determined to consist of Proteobacteria, Actinobacteria, Bacteroidetes, and Firmicutes [46,47], which is consistent with our results. Bacteroidetes and Firmicutes are capable of degrading dietary fiber, resistant starch, and other macromolecule carbohydrates in the host and then producing large quantities of short-chain fatty acids, such as acetic, butyric, and propionic acids [48]. These beneficial metabolites not only help to maintain an acidic environment in the intestine but also provide energy to the gut epithelial cells, defending the integrity of the intestinal barrier [49]. Supplementing bio-nanostructured selenium for lactating Barki sheep enriched the abundance of Bacteroidetes and had a positive effect on fibrinolytic bacteria propagation [50]. Similarly, in this study, the abundance of Bacteroidetes in the cats that that consumed the OTM diet was higher than in the cats that consumed the ITM diet. Meanwhile, at the family level, in the ITM group, the abundance of Ruminococcaceae and Lachnospiraceae was significantly lower than in the OTM group. Ruminococcaceae and Lachnospiraceae, the main flora in Firmicutes, can coexist with other beneficial bacteria and induce metabolites such as amino acids, vitamins, and short-chain fatty acids by breaking down complex carbohydrates and proteins [51,52]. In addition, it has been shown that Ruminococcaceae and Lachnospiraceae can restrain the concentration of pro-inflammatory cytokines as well as modulate intestinal immune homeostasis [53,54]. Our study demonstrated that OTMs promoted the proliferation of beneficial bacteria and then regulated the structure of gut microbiota by increasing the abundance and diversity, which ensured the intestinal health and overall health of cats.

The diversity and richness of microbiota is positively correlated with structural stability of intestinal ecological environment [55]. Stable gut microbiota structure helps beneficial bacteria compete for nutrients and colonization sites and is capable of inhibiting the growth and colonization of harmful pathogens as well as preventing the overpopulation of harmful bacteria [56,57]. A high diversity of gut microbiota helps to maintain intestinal barrier stability and immune system balance, preventing excessive inflammatory responses and autoimmune diseases [58,59]. Compared to the ITM group, we observed lower Simpson index and higher Shannon index in the OTM group. These results suggest that dietary OTMs contribute to a richer and more diverse microbial ecosystem, which is crucial for gut health.

Metabolites, as intermediates and products of metabolic pathways, have an essential function on the overall health and metabolic processes of organism. In our study, the fecal metabolites of cats were comprehensively analyzed to elucidate the impact of different trace mineral sources on the metabolic profile of cats. In total, 504 differential metabolites were identified in the ITM and OTM groups, such as 3-hydroxyoctadecanoylcarnitine, cervonoyl ethanolamide, linoleoyl ethanolamide, and chenodeoxycholyltryptophan, which were significantly up-regulated in the OTM group. 3-Hydroxyoctadecanoylcarnitine can be engaged in the β-oxidation of long-chain fatty acid as an intermediate in the fatty acid oxidation pathway [60]. Cervonoyl ethanolamide and linoleoyl ethanolamide are endogenous fatty amides belonging to the N-acylethanolamine family. These metabolites can inhibit the NF-κB signaling pathway, thereby reducing the release of cytokines and relieving inflammatory responses [61]. Cervonoyl ethanolamide also affects cellular lipid metabolism pathway by stimulating peroxisome proliferator-activated receptor γ [62]. Linoleoyl ethanolamide promotes insulin secretion through binding to the glucose-dependent insulinotropic receptor 119, thereby regulating the lipid metabolism and signal transduction pathways [63]. Chenodeoxycholyltryptophan is a specific bile acid–amino acid conjugate that may affect the bile acid synthesis and regulate the cholesterol metabolism pathway by activating the Takeda G-protein-coupled receptor 5 [64]. Although these metabolites have different modes of action in animals, they are all related to fatty acid metabolism pathway and bile acid metabolism pathway to a certain extent, which in turn affect the digestive system and immune system of animals. In our study, the correlation analysis between differentiated metabolites and microbial genera further supports the interplay between the gut microbiota and metabolite production. Our study identified 30 metabolites including agavoside A, madlongiside C, ganglioside GM2 (d18:1/23:0), simonin IV, ganglioside GM3 (d18:0/20:0), solamargine, 3-hydroxyoctadecanoylcarnitine, cervonoyl ethanolamide, linoleoyl ethanolamide, and chenodeoxycholyltryptophan that showed significant correlations with specific microbial genera, suggesting that changes in gut microbiota composition due to different trace mineral sources can directly influence the metabolite profile. This interplay underscores the importance of dietary OTMs in maintaining a balanced gut microbiota to support optimal metabolic function and overall health.

## 5. Conclusions

In conclusion, OTMs in feline diets affected promising improvements in immune function, inflammatory response, intestinal barrier integrity, gut microbiota composition, and metabolic health compared to ITMs. These findings highlight the potential benefits of organic trace minerals in promoting overall health and wellbeing in cats, suggesting that pet food formulations should consider these superior sources to enhance health outcomes in cats.

## Figures and Tables

**Table 3:** Fecal scoring criteria.

Score	Criteria
1	“Bullet like”, disintegrates with minimal pressure The stool is hard and dry, and it fractures when compressed
2	Properly shaped, it does not leave any residue when lifted Well-formed with slightly damp surface, leaves a trace when lifted
3	Moist and starting to lose its shape, it leaves a clear mark when lifted Very moist, retaining some definite form
4	Most or all structure is lost, lacking any definite shape Liquid stool with minimal consistency
5	Completely liquid stool

**Table 4:** BW, ADG and fecal score of cats.

Items	ITM	ITM + OTM	OTM	*p*-Valves
BW at d 0, kg	4.02 ± 0.08	3.88 ± 0.20	3.39 ± 0.32	0.14
BW at d 28, kg	3.92 ± 0.13	3.80 ± 0.22	3.41 ± 0.35	0.34
ADG, g/d	−3.75 ± 4.08	−3.10 ± 2.30	0.42 ± 4.81	0.72
Fecal score	1.95 ± 0.08	2.05 ± 0.10	2.08 ± 0.06	0.50

(1) ITM group: In the basal diet, the mineral premix was inorganic. (2) ITM + OTM group: In the basal diet, the mineral premix included 50% inorganic trace minerals and 50% organic trace minerals. (3) OTM group: In the basal diet, the mineral premix was organic. Values are presented as means ± SEMs, *n* = 6.

## Data Availability

Data are contained within the article.
